# Intravenous allogeneic umbilical cord blood–derived mesenchymal stem cell therapy in recessive dystrophic epidermolysis bullosa patients

**DOI:** 10.1172/jci.insight.143606

**Published:** 2021-01-25

**Authors:** Sang Eun Lee, Seung-Ju Lee, Song-Ee Kim, Kinam Kim, Boyoung Cho, Kyounghwan Roh, Soo-Chan Kim

**Affiliations:** 1 Department of Dermatology and Cutaneous Biology Research Institute, Gangnam Severance Hospital, Yonsei University College of Medicine, Seoul, South Korea.; 2 Cellular Therapeutics Team, Daewoong Pharmaceutical Co. Ltd., Seoul, South Korea.; 3 Department of Clinical Development, Kangstem Biotech Co. Ltd., Seoul, South Korea.; 4 Department of Dermatology, Yongin Severance Hospital, Yonsei University College of Medicine, Yongin, South Korea.

**Keywords:** Clinical Trials, Dermatology, Genetic diseases, Skin, Stem cell transplantation

## Abstract

**BACKGROUND:**

Recessive dystrophic epidermolysis bullosa (RDEB) is an incurable disease that causes severe mucocutaneous fragility due to mutations in *COL7A1* (encoding type VII collagen [C7]). In this phase I/IIa trial, we evaluated the safety and possible clinical efficacy of intravenous infusion of allogeneic human umbilical cord blood–derived mesenchymal stem cells (hUCB-MSCs) in patients with RDEB.

**METHODS:**

Four adult and two pediatric patients with RDEB were treated with 3 intravenous injections of hUCB-MSCs (1 × 10^6^ to 3 × 10^6^ cells/kg) every 2 weeks and followed up for 8–24 months after treatment. The primary endpoint was safety. Secondary endpoints related to efficacy included clinical parameters, such as disease severity score, wound assessment, itch and pain score, and quality of life. C7 expression levels and inflammatory infiltrates in the skin, as well as serum levels of inflammatory markers and neuropeptides, were also assessed.

**RESULTS:**

Intravenous hUCB-MSC infusions were well tolerated, without serious adverse events. Improvements in the Birmingham Epidermolysis Bullosa Severity Score, body surface area involvement, blister counts, pain, pruritus, and quality of life were observed with maximal effects at 56–112 days after treatment. hUCB-MSC administration induced M2 macrophage polarization and reduced mast cell infiltration in RDEB skin. Serum levels of substance P were decreased after therapy. Increased C7 expression was observed at the dermoepidermal junction in 1 of 6 patients at day 56.

**CONCLUSION:**

To the best of our knowledge, this is the first clinical trial of systemic administration of allogeneic hUCB-MSCs in patients with RDEB, demonstrating safety and transient clinical benefits.

**TRIAL REGISTRATION:**

ClinicalTrials.gov NCT04520022.

**FUNDING:**

This work was supported by Daewoong Pharmaceutical Co. Ltd. and Kangstem Biotech Co. Ltd.

## Introduction

Epidermolysis bullosa (EB) is a group of genetic diseases characterized by mechanical fragility of skin and mucosa (1). Recessive dystrophic EB (RDEB) is caused by mutations in *COL7A1*, which encodes type VII collagen (C7), the main constituent of anchoring fibrils at the dermoepidermal junction (DEJ). RDEB is one of the most severe forms of EB; it is characterized by recurrent blistering, chronic wounds, disabling scarring in the skin, and mucosa and internal organ dysfunctions, leading to substantial morbidity and mortality (2–4). Currently, there is no cure for this severe subtype of EB; however, novel therapeutic strategies have been developed in the fields of gene and cell therapies (5–15).

Mesenchymal stem cells (MSCs) have been identified as an attractive option for allogeneic cell therapy for RDEB based on their potential mechanisms of action, including immunomodulation, migration to damaged tissue, stimulation of tissue regeneration, and reduction of fibrosis, mainly through paracrine activities (8–11, 14–16). Locally injected allogeneic bone marrow–derived MSCs (BM-MSCs) have shown to accelerate wound healing, with transient C7 restoration in patients with RDEB and a mouse model of dystrophic EB (10). Two early-phase clinical trials of systemic administration of allogeneic BM-MSCs in 23 pediatric patients with RDEB reported variable clinical benefits that lasted for several months with satisfactory safety (8, 9). An additional recently published phase I/II trial of intravenous BM-MSC injection in 10 adult patients with RDEB also showed transient, but clinically meaningful, improvements in disease severity, skin inflammation, and pruritus, with no serious adverse events (AEs) (14).

To our knowledge, previous clinical trials for RDEB have examined the potential of BM-MSCs (8, 9, 14). However, umbilical cord blood (UCB) has become an attractive source of stem cells, because of its noninvasive collection procedure and rapid availability from cord blood banking (17, 18). Human UCB-derived MSCs (hUCB-MSCs) exhibit higher proliferation capacity and lower immunogenicity compared with BM-MSCs (17, 19). Data from a few reports support that UCB-MSCs may have greater immunosuppressive potential than other sources of MSCs (17–22). In addition, hUCB-MSCs have shown greater immunosuppressive and regenerative potential than BM- or peripheral blood–derived MSCs in murine wounding model (23). A preclinical study has demonstrated that repeated systemic infusions of human UCB-derived unrestricted somatic stem cells, a subpopulation of nonhematopoietic stromal stem cells, significantly extended the life span and reduced blistering in a RDEB mouse model (16). Given the promising results of the preclinical study, we conducted a first-in-human, phase I/IIa clinical trial of intravenous administrations of allogeneic hUCB-MSCs in patients with RDEB to determine safety, tolerability, and potential efficacy. We also analyzed changes in serum inflammatory markers, neuropeptides, and skin inflammatory infiltrates as well as C7 expression following hUCB-MSC treatment.

## Results

### Patient characteristics.

**Figure 1:**
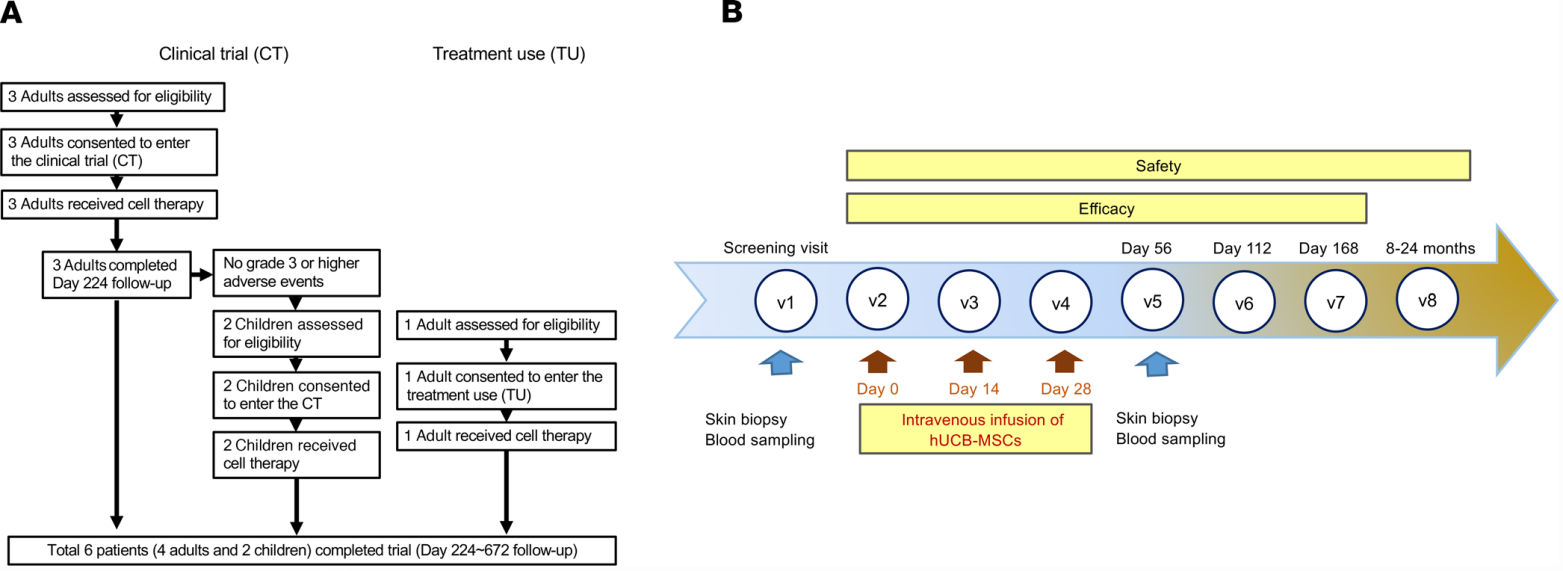
Study design. (**A**) Flow chart for clinical trial and treatment use (expanded access to investigational drugs for treatment use). (**B**) Study design for hUCB-MSC treatment and evaluation.

Between October 2016 and May 2019, 6 patients with RDEB were assessed for eligibility. Three adult and two pediatric patients were sequentially enrolled in the trial and received 3 repeated intravenous hUCB-MSC injections. One additional adult patient was treated with the same investigational product under the treatment use approval from the Korea Food & Drug Administration (KFDA), because they were too late for trial enrollment (Figure 1). All patients had moderate-to-severe or severe phenotypes, with various extracutaneous symptoms. Negative or markedly decreased expression of C7 noncollagenous-1 domain was found in baseline skin biopsies. Analysis of circulating autoantibodies against C7 using indirect immunofluorescence (IIF) was negative for all patients (Table 1). All adult patients received 3 × 10^6^ hUCB-MSCs/kg every 2 weeks, whereas the 2 pediatric patients received 1 × 10^6^ to 2 × 10^6^ hUCB-MSCs/kg every 2 weeks. All patients were carefully observed for clinical signs and laboratory test results related to potential thromboembolic events were monitored, even though a recent meta-analysis of randomized controlled trials reported no significant increase in the risk of thromboembolic events for patients treated with MSCs as compared with the control group (24). Demographics and clinical characteristics of participants and trial flow are provided in Figure 1 and Table 1. All patients completed at least 8 months (8–24 months) of follow-up after the first infusion.

### Safety.

AEs during the study period are summarized in Supplemental Tables 3 and 4 (supplemental material available online with this article; https://doi.org/10.1172/jci.insight.143606DS1). Overall, 50% of the patients treated reported ≥1 AE. The most frequent AE was wound infection (4 of 13 AEs, 30.8%), but all wound infections were thought to be due to the underlying RDEB. Only acute gastritis was considered as an AE determined to be possibly related to cell therapy. No severe AEs (defined by Common Terminology Criteria for Adverse Events) at grade 3 or higher were reported, suggesting that intravenous hUCB-MSC injections were generally well tolerated. There were no clinically significant changes in laboratory test values, except increased basal levels of C-reactive protein (CRP) and fibrinogen, vital signs or electrocardiogram results during the study period. There were no changes in tissue-bound immunoreactants using IIF following cell therapy.

### Clinical efficacy.

**Figure 2:**
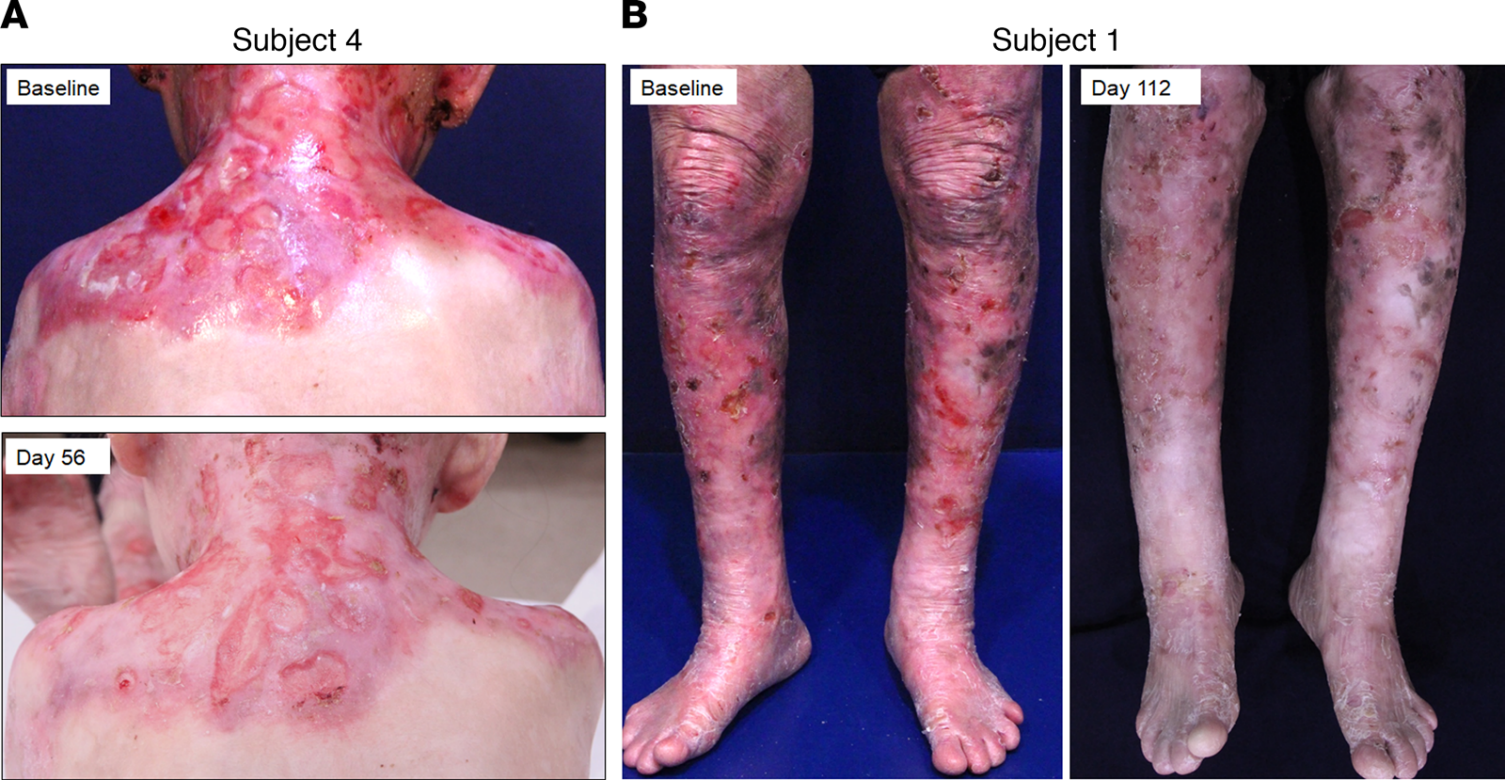
Marked reduction in erythema and erosions after hUCB-MSC treatment. Photographs of a pediatric (**A**, subject 4) and an adult patient with RDEB (**B**, subject 1) at baseline and after 3 repeated injections of hUCB-MSCs.

**Figure 3:**
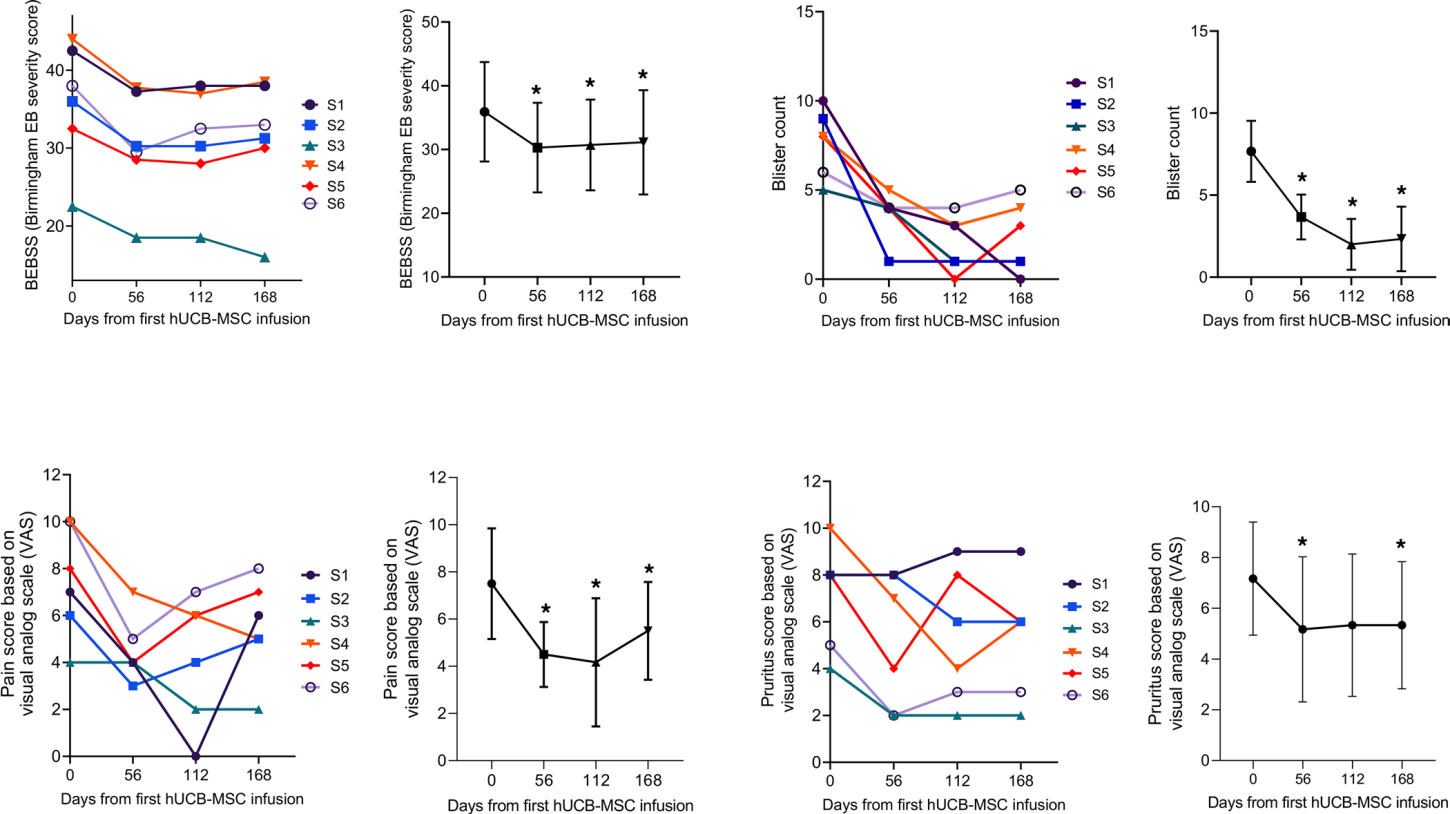
Systemic treatment with hUCB-MSCs improved clinical symptoms in patients with RDEB. The time course of changes in disease severity (assessed by Birmingham Epidermolysis Bullosa Severity Score [BEBSS]), blister count, visual analog scale (VAS) pain score, and VAS pruritus score was assessed throughout the trial. For each parameter, a graphical representation of mean score per visit with range per visit was added. Two-tailed Student’s *t* test was performed for all the comparisons (*n* = 6). *P < 0.05. S, subject. Values are shown as the mean ± SEM.

hUCB-MSC treatment markedly reduced erythema and erosions in patients with RDEB (Figure 2). At day 56, the mean clinical severity scores assessed by the Birmingham Epidermolysis Bullosa Severity Score (BEBSS) and total body surface area (TBSA) affected by RDEB significantly decreased by 5.6 points (95% CI, –7.39 to –3.86) and 5.4 points (95% CI, –8.14 to –2.61), respectively. Blister count and the ratio of blister area to body surface area also decreased by 4 points (95% CI, –6.74 to –1.26) and 2 points (95% CI, –4.02 to –0.06), respectively, at day 56 compared with baseline. After day 56, these clinical effects of hUCB-MSCs were either maintained or slightly attenuated over time until day 168 (Figure 3 and Supplemental Figure 1). Chronic nonhealing wounds in RDEB are associated with decreased quality of life (QOL) and increased risk of cutaneous squamous cell carcinoma (cSCC). We evaluated the effect of hUCB-MSCs on the healing of chronic open wounds that were unhealed for at least 12 weeks with wound size >100 cm^2^, as defined in a previous study (13). One pediatric (subject 4) and one adult (subject 6) subject each had 2 chronic open wounds. Of the 4 chronic wounds from 2 subjects, 2 wounds (1 from subject 4 and 1 from subject 6) (50%) showed a 50% or greater reduction in wound size compared with baseline at day 56. Of these 2 wounds, only 1 remained at least 50% healed by day 112.

hUCB-MSC treatment resulted in a substantial mean reduction in pain (−3 points on visual analogue scale [VAS] score, 95% CI, –4.76 to –1.24) and itch (−2 points on VAS score, 95% CI, –3.76 to –0.24) from baseline to day 56. Mean VAS scores for pruritus were maintained by day 168, while pain VAS scores showed a gradual increase over time (Figure 3). At day 56, QOL, as assessed by a QOL in EB questionnaire (QOLEB), was improved by 6.2 points (95% CI, –8.69 to –3.65). The baseline and mean change from the baseline for the secondary outcome data are summarized in Supplemental Table 5. As shown in Supplemental Figure 2, age subgroup analyses (children vs. adults) showed no significant between-group differences in the secondary outcomes, including BEBSS, TBSA, blister count and area, itch and pain scores, and QOLEB.

### Molecular assays for C7 in skin.

**Figure 4:**
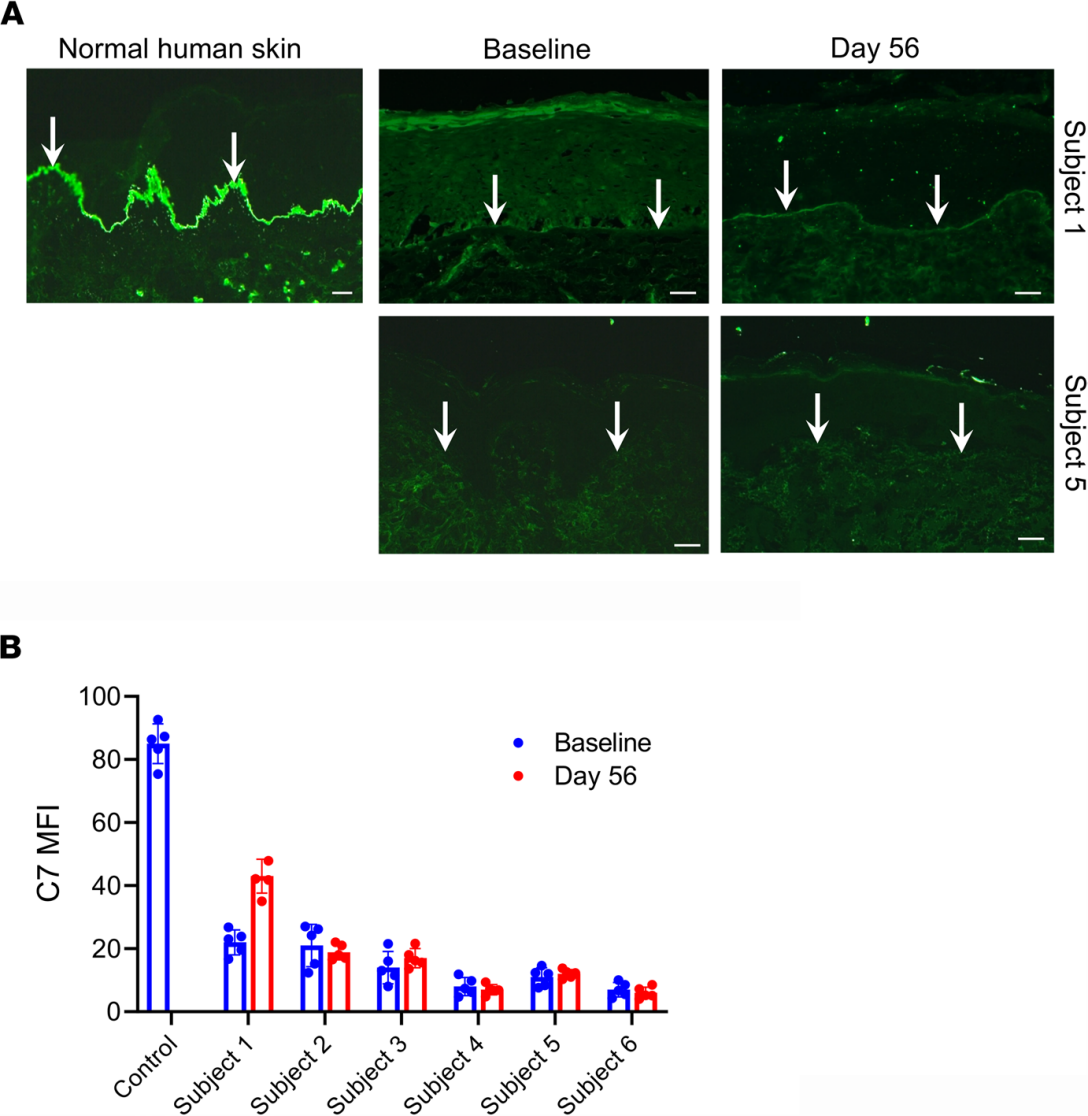
Systemic treatment with hUCB-MSCs does not significantly affect the expression levels of C7 at the DEJ in most patients, except for in 1 patient (subject 1), who showed an increase in C7 expression at day 56. (**A**) Representative immunofluorescence staining for type VII collagen (C7) using LH7.2, a monoclonal antibody that recognizes the NC1 domain of C7, on skin biopsy samples obtained before treatment (baseline) and at day 56 from patients with RDEB (subjects 1 and 5) receiving hUCB-MSC treatment. Scale bars: 20 μm. White arrows indicate C7 expression at the dermoepidermal junction (DEJ). (**B**) The intensity of staining for C7 expression along the DEJ was morphometrically quantitated as MFI using ImageJ (NIH). Values are shown as the mean ± SEM.

Then we evaluated whether systemic infusions of hUCB-MSCs could restore C7 and anchoring fibrils in RDEB skin by immunofluorescence staining and transmission electron microscopy (TEM) analysis of skin of patients before and after treatment. On day 56, 1 patient (subject 1) showed an increase in C7 expression levels at the DEJ, as assessed by mean fluorescence intensity (MFI) compared with baseline, while others (subjects 2–6) showed no significant changes in C7 expression in skin after MSC treatment (Figure 4). No obvious differences in anchoring fibril structure or distribution were observed between baseline and day 56 in all 6 patients, as assessed by TEM (data not shown).

### Changes in skin infiltration of macrophages and mast cells.

**Figure 5:**
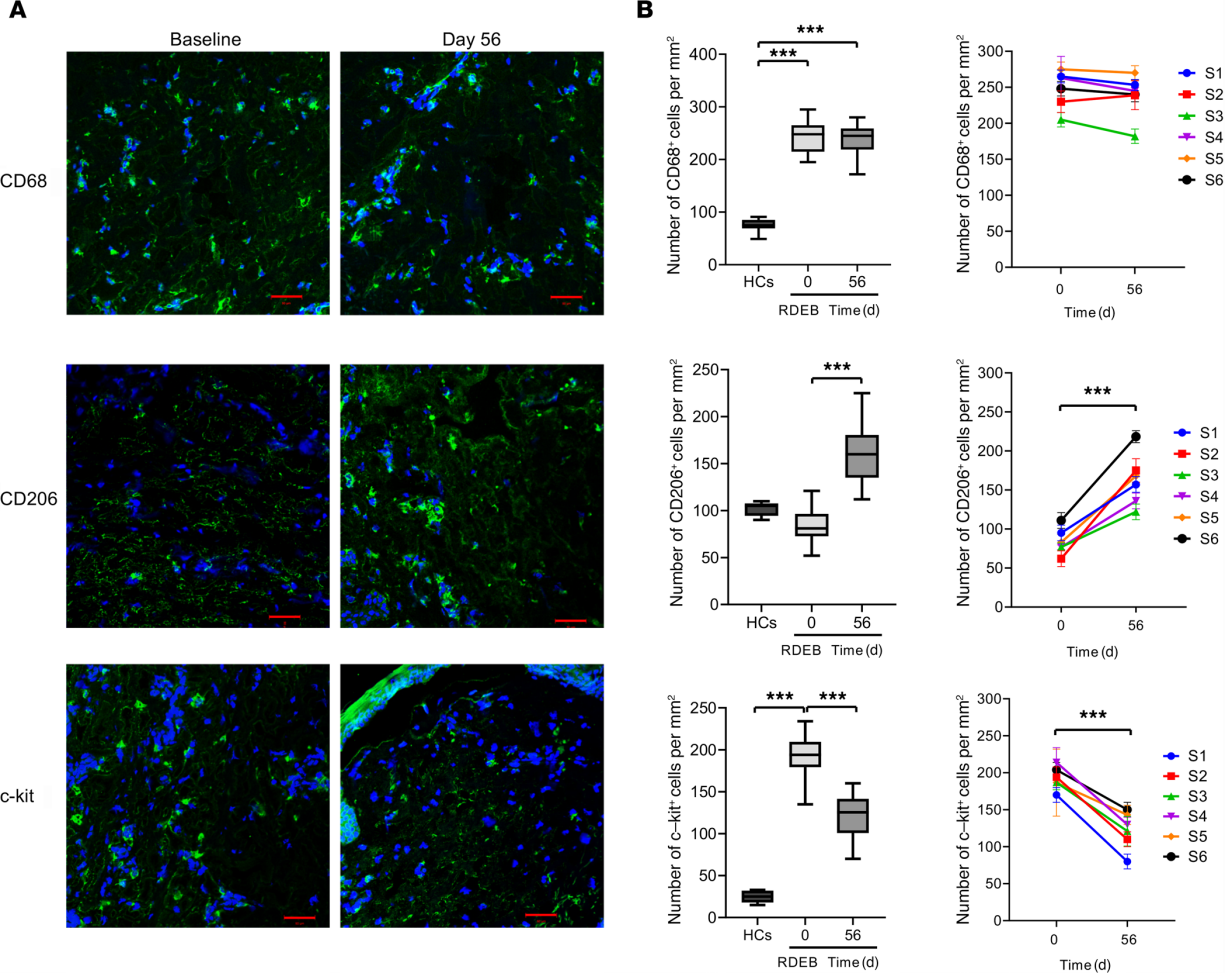
hUCB-MSC treatment modulates macrophage phenotype and mast cell infiltration in skin from patients with RDEB. (**A**) Representative immunofluorescence staining for total macrophages (CD68), CD206^+^ macrophages, and mast cells (c-kit) on skin biopsy samples before treatment (baseline) and at day 56 for 6 matched pairs of patients with RDEB receiving hUCB-MSC treatment. Scale bars: 50 μm. (**B**) Mean total numbers of skin-infiltrating cells in biopsies from healthy controls (HCs) and RDEB skin at day 0 and at day 56 following hUCB-MSC treatment. By day 56, hUCB-MSC treatment markedly increased CD206^+^ macrophage counts and reduced mast cell counts. Values are shown as the mean ± SEM. Wilcoxon’s signed-rank test was performed for all the comparisons (*n* = 6). ***P < 0.001. S, subject.

Macrophages have a central role in maintaining tissue homeostasis and repair. Classic proinflammatory (M1) and alternatively activated, antiinflammatory (M2) macrophages exhibit distinct phenotypes and functions (25, 26). Previous studies indicated that MSCs can promote M2 polarization of tissue macrophages, contributing to tissue regeneration (27–29). Therefore, we analyzed the phenotypes of macrophages in the skin of patients with RDEB before and after hUCB-MSC treatment. The number of CD68^+^ total macrophages was higher in the skin of patients with RDEB at baseline than in healthy controls. Intravenous administration of hUCB-MSCs did not affect the density of CD68^+^ total macrophages but significantly increased macrophages expressing CD206, a marker of M2 macrophages, in RDEB skin at day 56 (Figure 5). Mast cells play a central role in neuroinflammatory pain and itch (30, 31). Baseline skin biopsies of patients with RDEB showed a significant increase of mast cell infiltration compared with normal human skin, but mast cell infiltration was significantly reduced 56 days after hUCB-MSC treatment (Figure 5).

### Changes in systemic inflammatory markers and neuropeptides.

**Figure 6:**
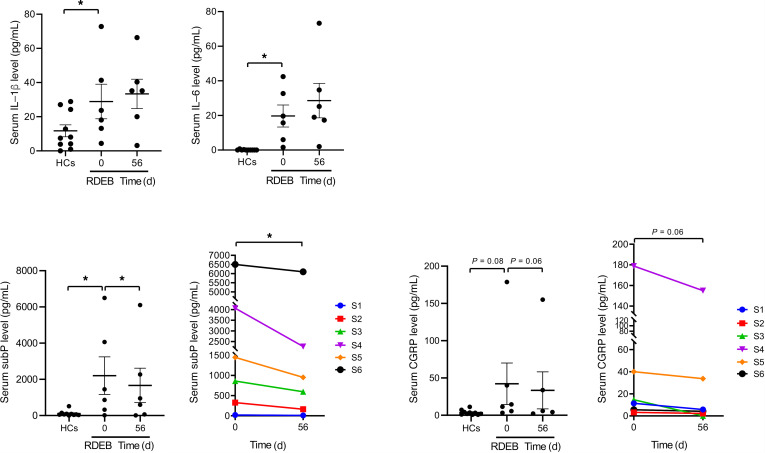
hUCB-MSC treatment reduces serum substance P levels in patients with RDEB. Serum levels of inflammatory cytokines (IL-1β and IL-6) and neuropeptides (substance P and CGRP) were assessed in healthy controls (HCs) and patients with RDEB (*n* = 6) at baseline and at day 56 following hUCB-MSC treatment. Values are shown as the mean ± SEM. Wilcoxon’s signed-rank test was used to assess the statistical difference between the repeated measurements in the same patient. *P < 0.05. S, subject.

Chronic wounds in RDEB trigger systemic inflammation that may contribute to multiple-organ damage (2, 3, 32–34). Since MSCs have potent immunomodulatory capacities, we investigated the effect of hUCB-MSC infusion on serum inflammatory markers in patients with RDEB. Serum levels of CRP fluctuated in individual patients over time, but no significant change in the mean CRP values was observed 56 days after hUCB-MSC treatment (data not shown). Additionally, baseline serum levels of proinflammatory cytokines, IL-1β and IL-6, were elevated in patients with RDEB compared with those in healthy controls, but these levels were not significantly altered by hUCB-MSC treatment on day 56 (Figure 6).

Given the remarkable efficacy of hUCB-MSC treatment in reducing pain and itch in patients with RDEB in this study, we also analyzed the changes in serum levels of neuropeptides. Baseline serum levels of substance P were significantly higher in patients with RDEB compared with age-matched healthy control values, and, notably, substance P levels were significantly reduced 56 days after hUCB-MSC treatment. Serum calcitonin gene–related peptide (CGRP) levels were also higher in patients with RDEB than in healthy controls, and these levels were reduced from baseline after hUCB-MSC treatment (*P* = 0.06), but these changes were not statistically significant (Figure 6).

## Discussion

This open-label, phase I/IIa clinical trial shows that 3 repeated intravenous administrations of allogeneic hUCB-MSCs are well tolerated and potentially provide clinical benefits by reducing disease severity, disease-affected body area, blister count, and pain and itch and improving QOL in children and adults with moderate-to-severe or severe RDEB. This study is meaningful in that it is the first clinical trial to our knowledge to apply MSCs derived from UCB, systemically, to patients with RDEB.

Three separate intravenous infusions of hUCB-MSCs did not cause serious AEs. A previous clinical trial of BM-MSCs in 10 adult patients with RDEB reported development of cSCC in 2 participants about 6–7 months after the injections (14), suggesting careful monitoring of this potential complication, particularly in adult patients. In this trial, of the 4 adult patients, 1 patient (subject 6) was followed up for 16 months, and the remaining 3 patients (subjects 1, 2, and 3) were followed up for up to 24 months; there was no development of cSCC during these follow-up periods. However, long-term follow-up data for more patients is needed to accurately evaluate the potential relationship between allogeneic hUCB-MSCs therapy and the risk of cSCC in RDEB.

Although the primary objective was to assess safety, our data provide evidence of the potential efficacy of hUCB-MSC therapy in various clinical aspects of RDEB. hUCB-MSC infusions significantly reduced disease severity, as assessed by using BEBSS, affected body surface area, blister count, and blister area, with a maximal effect at day 56 in most patients. Over time, these clinical effects of hUCB-MSCs were progressively attenuated, but some patients showed sustained improvement up to day 168. With regard to wound healing, 50% (2 of 4) of large open wounds that were present for at least 12 weeks achieved 50% or greater healing by 56 days after treatment. Despite the small number of available chronic large wounds and the lack of control wounds, based on a previous report that indicated that a 50% reduction in chronic RDEB wounds is clinically meaningful in terms of improvement in patient-reported outcomes (13), our results indicate that hUCB-MSC therapy exerts beneficial effects on wound healing in RDEB. In addition to the improvement of cutaneous lesions, intravenous administration of hUCB-MSCs also relieved the symptom of dysphagia in 1 patient (subject 3), allowing the scheduled balloon dilation for esophageal stricture to be delayed.

Recalcitrant pain and pruritus are among the most bothersome symptoms of RDEB (35–38). Pain in severe generalized RDEB is often very severe in that it does not respond well to potent opioid analgesics, and its intensity was shown to be greater than in postherpetic neuralgia (39). RDEB also causes severe pruritus that is thought to be associated with cutaneous inflammation secondary to barrier disruption, wound healing processes, and dysregulated activity of epidermal nerve fibers (38).

In this study, hUCB-MSCs markedly reduced pain and pruritus in patients with RDEB by reducing average VAS scores by 3 cm and 2 cm on day 56, respectively. Given that the minimum important difference for clinical improvement of chronic pain or pruritus has shown to be 2 to 3 cm on the VAS score (40, 41), our data suggest that hUCB-MSC treatment is effective in achieving a clinically relevant improvement in pain and pruritus in RDEB that may lead to improved QOL.

When comparing the clinical efficacy of hUCB-MSCs with that of BM-MSCs in RDEB, the mean differences in BEBSS and QOLEB scores at day 56 in our study were 5.6 points (95% CI, –7.39 to –3.86) and 6.2 points (95% CI, –8.69 to –3.65), which were comparable to those in previous studies using BM-MSCs in children (mean difference of BEBSS at day 60 was 5.2, QOLEB score was 4.4) and adults (mean difference of BEBSS at day 60 was 1.61, QOLEB score was 3.13) (8, 14). These findings indicate that hUCB-MSCs provide comparable therapeutic effects to BM-MSCs in improving disease severity and QOL in patients with RDEB. Regarding the itch and pain outcome, it is difficult to directly compare the effect of hUCB-MSCs with that of BM-MSCs because of the different measurement tools in each study and the lack of data on pain in the previous study using BM-MSCs in adult patients. The results of our trial show that hUCB-MSCs effectively ameliorate pain as well as pruritus in both children and adults with RDEB.

When comparing the clinical efficacy of allogeneic MSCs in pediatric and adult patients with RDEB, previous studies reported a better clinical efficacy of BM-MSCs in children (mean difference of BEBSS at day 60 was 5.2) than in adults (mean difference of BEBSS at day 60 was 1.61) (8, 14), which was speculated to be associated with more severe systemic inflammation and scarring in adults patients with RDEB. In contrast, the therapeutic efficacy of hUCB-MSCs in this trial (mean difference of BEBSS at day 56 was 5.13 in children and 7.18 in adults) was similar in children and adults. Considering that the number of cells administered per kilogram of patient’s body weight was lower in children (3 infusions, each dose 1 × 10^6^ to 2 × 10^6^ cells/kg) than in adults (3 infusions, each dose 3 × 10^6^ cells/kg) in this trial, additional clinical data are needed to accurately compare the effects of hUCB-MSCs in pediatric and adult patients with RDEB.

Mechanistically, systemic treatment with hUCB-MSCs did not restore the expression of C7 and anchoring fibril at the basement membrane in the skin of most patients, except for 1, who showed increased C7 expression on day 56. These findings are consistent with previous clinical trials of systemic administration of BM-MSCs (8, 9, 14) and suggest that the therapeutic benefits of hUCB-MSCs are not primarily caused by the recovery of C7 expression.

The mechanisms underlying hUCB-MSC–mediated therapeutic effects on RDEB are still unknown. To understand their mechanisms of action, we further assessed the changes in blood biomarkers of inflammation and innate immune cells infiltration in the skin following hUCB-MSC treatment.

Patients with RDEB showed higher serum levels of CRP and proinflammatory cytokines, IL-1β and IL-6, compared with healthy controls, suggesting systemic inflammation in severe generalized RDEB. Despite the reductions in disease severity and cutaneous erythema, serum levels of CRP, IL-1β, and IL-6 showed no significant change after hUCB-MSC treatment compared with baseline, suggesting that these inflammatory molecules are not suitable biomarkers for monitoring therapeutic response to hUCB-MSCs. CRP and IL-6 are markers of acute phase response, and fluctuating CRP values in individual patients might reflect the dynamic inflammatory status in patients with RDEB. Our findings are consistent with those of a prior clinical trial of BM-MSCs in patients with RDEB, which reported that inflammatory molecules were generally unchanged, but high mobility group box-1 was significantly decreased after treatment (14, 32).

To date, little is known about the pathophysiological mechanism of pain and pruritus in RDEB, but recent study found a decreased nerve fiber density and increased number of activated mast cells in skin of patients with RDEB, indicating neuropathic pain and itch (42, 43). Sensory nerve-derived neuropeptides, substance P, and CGRP participate in neuroimmune crosstalk, thereby leading to neurogenic inflammation, neuropathic pain, and itch (30, 31). Moreover, the substance P-neurokinin 1 receptor antagonists have been reported to effectively reduce pruritus in patients with prurigo (44), cutaneous T cell lymphoma (45), and EB (46).

Interestingly, serum substance P levels were significantly higher and serum CGRP levels tended to be higher in patients with RDEB compared with those in healthy controls. In addition, serum substance P and CGRP levels were reduced after hUCB-MSC treatment. Consistent with a previous study (42), increased numbers of mast cells were detected in the skin of patients with RDEB compared with healthy skin at baseline. Of note, infiltration of mast cells was substantially reduced after hUCB-MSC treatment. These findings suggest the possible role of substance P and mast cells in the neuropathic pain and itch in patients with RDEB. Furthermore, the effective attenuation of pain and pruritus in hUCB-MSC–treated patients with RDEB could be due to the inhibition of substance P levels and mast cell activation.

Another interesting aspect of this work is the evaluation of changes in macrophage phenotype following hUCB-MSC treatment. Macrophages play an important role in immune modulation, tissue repair, and fibrosis (25, 26). In this study, we found that hUCB-MSC treatment did not alter the number of total macrophages but markedly increased M2 macrophage infiltration in the skin of patients with RDEB. These findings are consistent with observations from a preclinical study of human UCB-derived nonhematopoietic stromal stem cells in RDEB mouse model (16), supporting that hUCB-MSC therapy–induced M2 polarization of tissue macrophages also occurs in patients with RDEB. The increase in these prorepair or alternatively activated M2 macrophages might contribute to accelerated wound healing and the resolution of inflammation following hUCB-MSC treatment; however, further studies are necessary to elucidate the functional significance of macrophage M2 polarization.

In addition to studies of allogeneic MSCs from UCB or bone marrow, there has been an ongoing clinical trial using allogenic ABCB5-expressing MSCs (ABCB5^+^ MSCs) in patients with RDEB (ClinicalTrials.gov NCT03529877) since January 2019. Recently, human dermal ABCB5^+^ MSCs have emerged as a promising novel therapeutic candidate for the treatment of various incurable diseases, with their immunomodulatory effects and safety (47). In addition, there is growing evidence that MSC-derived extracellular vesicles augment the therapeutic potential of MSCs in various pathways (48, 49). Especially in RDEB, MSC-derived extracellular vesicles can support the transport of C7 within the extracellular space and provide fibroblasts with mRNA encoding C7 (50). Taken together, in addition to hUCB-MSCs and BM-MSCs, human dermal ABCB5^+^ MSCs or MSC-derived extracellular vesicles also can be alternative therapeutic candidates in the field of cell therapy for RDEB.

The limitations of this open-label study included the small number of patients and the lack of a control placebo-treated arm.

In conclusion, allogeneic hUCB-MSCs were well tolerated when administered intravenously 3 times in both pediatric and adult patients with RDEB. hUCB-MSC therapy reduced disease severity, with significant improvements noted in erythema in the affected area, blister count, pain, pruritus, and QOL. In addition, transient clinical benefits of allogenic hUCB-MSCs were observed, with a maximal efficacy at 56–112 days after treatment and a gradual attenuation of these clinical benefits through day 168. In the future, larger clinical trials are needed to investigate the optimal dosage, number of injections, differential efficacy of different tissue-derived MSCs, and the long-term safety of allogeneic MSC therapy for RDEB.

## Methods

### Patients, study design, and procedures.

This phase I/IIa, single-center, nonrandomized, open-label trial to evaluate the safety and efficacy of hUCB-MSCs for patients with RDEB was conducted at Gangnam Severance Hospital. The diagnosis of RDEB was made by immunofluorescence antigen mapping, TEM, and mutation analysis of the *COL7A1* gene. Detailed criteria for patient recruitment are described in Supplemental Table 1. Patients who provided informed consent were screened within 4 weeks before the start of the cell therapy. The visit schedule consisted of a 4-month run-in period that included a screening visit and an enrollment visit, 3 administrations of hUCB-MSCs, and an 8- to 24-month follow-up period (Supplemental Table 2). Patients received 3 separate intravenous injections of hUCB-MSCs every 2 weeks and then were assessed at days 56, 112, and 168 and 8–24 weeks after treatment. Following the first administration of hUCB-MSCs, the patients remained hospitalized for 24 hours for observation of possible AEs. Peripheral blood samples were obtained at each visit for safety laboratory tests and biomarkers analysis. Skin biopsy samples obtained at visits 1 and 5 were examined for changes in C7 and anchoring fibril expression by immunofluorescence staining and TEM, respectively, and skin infiltration of immune cells after treatment. Study design, with schedules, is described in Supplemental Table 2.

### Production of hUCB-MSCs.

hUCB-MSCs were manufactured and expanded according to good manufacturing practice (GMP) regulations. hUCB-MSCs from UCB of healthy donors were isolated and expanded with the KSB-3 Complete Medium Kit (Kangstem Biotech Co. Ltd.) at the GMP facility of Kangstem Biotech Co. Ltd. The manufactured cells were confirmed to meet the quality control criteria approved by the Ministry of Food and Drug Safety.

### Outcome measures.

The primary endpoints of the investigation were the safety and tolerability of 3 separate intravenous administrations of hUCB-MSCs. Safety was assessed through the monitoring of AEs, laboratory assessments, vital signs, electrocardiograms, and abbreviated physical examinations at each visit during the 8- to 24-month period after treatment. Secondary efficacy endpoints included (a) disease severity scores assessed by BEBSS and TBSA affected by EB; (b) wound assessment by clinical photograph, blister count, and the ratio of blister area to body surface area; (3) VAS for pain and pruritus; and (4) QOLEB during the 6-month period after treatment compared with those in the screening period.

### Immunofluorescence staining and TEM analysis.

Frozen skin tissues from the patients were sectioned at 5 μm and stained with primary antibodies, including mouse monoclonal [LH7.2] antibodies against C7 (ab6312; Abcam), mouse monoclonal antibodies against CD206 (321102, Biolegend), mouse monoclonal antibodies against CD68 (ab955, Abcam), and rabbit polyclonal antibodies against c-kit (A4502, Dako). Alexa Fluor 488–conjugated rabbit anti-mouse IgG and goat anti-rabbit IgG (both from Thermo Fisher Scientific) were used as secondary antibodies. Sections were stained with DAPI (Thermo Fisher Scientific). Images were captured using an LSM 780 confocal microscope (Carl Zeiss). Negative controls omitting the primary antibody were also performed (data not shown). The skin tissue sections were fixed in Karnovsky’s fixative and examined under a TEM (H-7600, Hitachi).

### MFI.

MFI was calculated for each immunofluorescence stained image for C7 using ImageJ (NIH). Five measurements were taken at regular intervals using 8 × 8 pixels every 100 pixels along the dermal-epidermal junction. The values are presented as mean ± SEM.

### IIF.

The detection of circulating anti-C7 autoantibodies was performed in all patients by an IIF study performed on salt-split normal human skin substrate. To evaluate the presence of anti-C7 antibodies, patient serum was obtained at baseline, day 56, day 112, and day 168 for evaluation of anti-C7 antibodies by salt-split IIF.

### Serum biomarkers measurement.

Levels of biomarkers were measured in pretreatment and day 56 serum samples. IL-1β (Human IL-1β/IL-1F2 Quantikine ELISA Kit, DLB50, R&D Systems), IL-6 (Quantikine ELISA Kit, D6050, R&D Systems), substance P (Substance P Parameter Assay Kit, KGE007, R&D Systems), and CGRP (human CGRP kit, A05481.96, Bertin Pharma) were quantified by individual competitive ELISAs according to the manufacturer’s instructions. The normal reference range for the proinflammatory cytokines and neuropeptides was defined from a population of 10 healthy subjects ranging from 18 to 50 years of age.

### Statistics.

In this trial, 6 patients were enrolled and completed the follow-up. For the secondary outcomes (clinical parameters), the mean differences from baseline were analyzed, together with a *P* value and a 95% CI (2-tailed paired *t* test). For the serum biomarkers and the number of cell infiltrates in the skin, Wilcoxon’s signed-rank test was used in a statistical analysis to compare the paired samples of patients before and at different time points during treatment. Two-tailed Student’s *t* test was used for comparing the secondary outcomes (clinical parameters) among age subgroups. Statistical analyses were performed using Prism 8 (GraphPad Prism). Statistical significance was defined as *P* < 0.05.

### Study approval.

All methods and procedures associated with this study were approved by the institutional review board (no. 3-2015-0285) of Yonsei University College of Medicine and were performed in compliance with the Declaration of Helsinki and good clinical practice, as defined under the KFDA regulations and the International Conference on Harmonisation guidelines. Prior to inclusion in this study, written informed consent was received from all participants or their guardians in case of pediatric patients. Written informed consent was provided for use of the patient photographs. This clinical trial began in October 2016 after being approved by the institutional review board of Gangnam Severance Hospital in December 2015 and by the KFDA in June 2016. This study was retrospectively registered on Clinicaltrials.gov, as institutional review board and KFDA approval is sufficient to initiate a clinical trial and registration on Clinicaltrials.gov is not mandatory in Korea. Our study includes all information, including demographics and results, for all patients.

## Author contributions

SCK and SEL designed the study and performed patient enrollment and patient care. SEL, SJL, and SCK prepared the manuscript. SEL, SJL, and SEK acquired data and tissue and performed laboratory experimentation and biostatistical analysis. KK and BC provided financial support. KR provided the investigational product (hUCB-MSCs).

## Supplementary Material

Supplemental data

Trial reporting checklists

ICMJE disclosure forms

## Figures and Tables

**Table 1:**
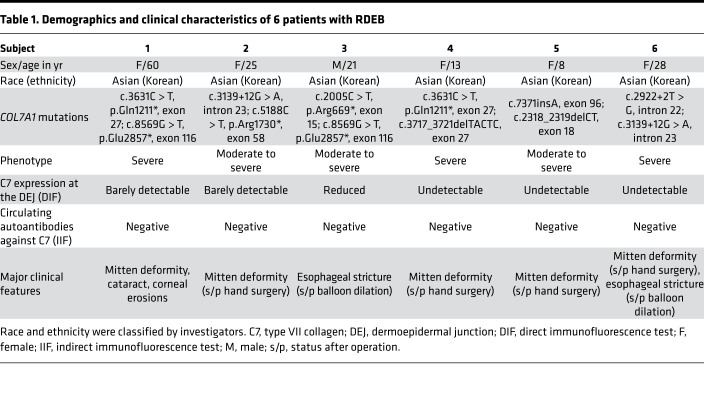
Demographics and clinical characteristics of 6 patients with RDEB

## Version 1 01/25/2021

Electronic publication
