# Supplementary material for: Intravenous allogeneic umbilical cord blood–derived mesenchymal stem cell therapy in recessive dystrophic epidermolysis bullosa patients
Source: JCI Insight. 2021 Jan 25;6(2):e143606. doi: 10.1172/jci.insight.143606 (PMC7934866; doi:10.1172/jci.insight.143606)
Supplement: Supplemental data [file jciinsight-6-143606-s175.pdf]

Supplemental Tables

Supplemental Table 1. Inclusion and Exclusion Criteria for Participants

**Inclusion criteria**
1. Subjects diagnosed as recessive dystrophic epidermolysis bullosa (RDEB) by immunofluorescence antigen mapping (partial or complete type VII collagen deficiency), transmission electron microscopy (partial or complete absence of anchoring fibrils), and mutation analysis for *COL7A1*.
2. Subjects who are ≥ 10 years and ≤ 60 years of age at the time of enrolment.
3. Adult subjects (≥ 19 years of age) willing to sign the informed consent form. Pediatric subjects (≥ 10 years and ≤ 18 years) whose legal parent/guardian has voluntarily signed the informed consent form.
**Exclusion criteria**
1. Subjects who have had other investigational drugs within 1 year before baseline visit
2. Subjects who have received immunotherapy including oral corticosteroids for ≥ 1 week or chemotherapy within 60 days of enrolment.
3. Subjects who have received all types of live vaccines except influenza vaccine within 30 days of enrolment.
4. Subjects who have congenital or acquired immunodeficiencies.
5. Subjects with a known allergy to any of the constituents of the investigational product.
6. Subjects with signs of active infection.
7. Subjects who have acute and chronic hepatitis with positive hepatitis B virus surface antigen, IgM or IgG antibody to hepatitis B core antigen.
8. Subjects with a history or evidence of malignancy, including cutaneous squamous cell carcinoma.
9. Subjects with positive indirect immunofluorescence (IIF) with binding to the base of salt split skin.
10. Pregnant or breast-feeding women or sexually active women of childbearing potential who are unwilling to practice contraception during the study

Supplemental Table 2. Detailed Study Design

Period	Screening	Treatment			Follow up			Post-Study Visit
Visit No.	V1	V2	V3	V4	V5	V6	V7	
Day (week)	-4 month ~ Day -7	Day 0	Day 14 (2w)	Day 28 (4w)	Day 56 (8w)	Day 112 (16w)	Day 168 (24w)	32w~96w
Screening evaluation and acquisition of written informed consent	●							
Past medical history	●							
Demographic survey	●							
Body measurements[1)], Vital signs[2)]	●	●	●	●	●	●	●	●
Pregnancy test [3)]	●							
Blood samples[4)]	●	●	●	●	●		●	●
Electrocardiogram	●	●	●	●				
Infusion of hUCB-MSCs		●	●	●				
Skin biopsy[5)]	●				●			
Disease severity skin score[6)]	●				●	●	●	
Wound assessment (clinical photograph, blister count)	●	●	●	●	●	●	●	
Visual analogue scale (VAS) for pain and pruritus	●	●	●	●	●	●	●	
Quality of life questionnaire	●				●	●	●	
Evaluation of adverse events[7)]		●	●	●	●	●	●	●
Investigation for combined medication		●	●	●	●	●	●	●

1 Height, weight, body mass index (BMI)

2 Blood pressure, pulse rate, body temperature

3 Urine HCG test

4 Laboratory test was performed only for full blood count, renal and liver profiles, and inflammatory markers. In addition, test for hepatitis B virus surface antigen, IgM or IgG antibody to hepatitis B core antigen, anti-HIV Ag/Ab, and anti-hepatitis C virus was performed only for the screening evaluation.

5 Immunofluorescent study and transmission electron microscopy

6 ① Birmingham Epidermolysis Bullosa Severity Score (BEBSS), ② Global Severity Score, ③ Blister area/body surface area

7 The adverse events were evaluated by observing the patient for 60 minutes (24 hours in visit 2) after administering the clinical drug. In the case of follow-up, adverse events were evaluated through physical measurements, physical examinations, and blood samples.

Supplemental Table 3. Summary of adverse events (AEs)

	Number (%)
**Total number of patients**	6 (100)
**Number of patients who experienced AEs**	3 (50)
**Total number of AEs**	13 (100)
**Grade[A]**	**Number of AEs (%)**
**Grade 1**	8 (61.5)
**Grade 2**	5 (38.5)
**Grade 3~5**	0 (0)
**Relationship to the study drug**	
**Definitely**	0 (0)
**Possibly**	1 (7.7)
**Likely**	0 (0)
**Unlikely**	1 (7.7)
**Not related**	11 (84.6)
**Outcome**	
**Resolved**	12 (92.3)
**Continuing**	1 (7.7)
**Action taken**	
**None**	3 (23.1)
**Required concomitant medication**	10 (76.9)

[A]Grading scales were measured according to Common Terminology Criteria for Adverse Events (CTCAE) version 5.0.

Supplemental Table 4. Adverse events (AEs) classified by systemic organ classes

		Relationship to the drug (n (%))				
System organ class	Adverse event	Definitely	Possibly	Likely	Unlikely	Not related
Generalized	General condition deterioration	0 (0)	0 (0)	0 (0)	0 (0)	1 (7.7)
	Dizziness	0 (0)	0 (0)	0 (0)	0 (0)	1 (7.7)
Gastrointestinal	Acute gastritis	0 (0)	0 (0)	1 (7.7)	0 (0)	0 (0)
	Nausea	0 (0)	0 (0)	0 (0)	0 (0)	1 (7.7)
Infection	Fever	0 (0)	0 (0)	0 (0)	0 (0)	1 (7.7)
	Herpes zoster	0 (0)	0 (0)	0 (0)	1 (7.7)	0 (0)
	Wound infection	0 (0)	0 (0)	0 (0)	0 (0)	4 (30.8)
	Upper respiratory tract infection	0 (0)	0 (0)	0 (0)	0 (0)	1 (7.7)
Ocular	Lacrimal duct obstruction	0 (0)	0 (0)	0 (0)	0 (0)	1 (7.7)
Ear, Nose, and Throat	Epistaxis	0 (0)	0 (0)	0 (0)	0 (0)	1 (7.7)

Supplemental Table 5. Secondary outcomes

Outcome	Baseline mean (SD)	Day 56 mean (SD)	Mean difference between Day 56 and Baseline (95% CI)	Day 168 mean (SD)	Mean difference between Day 168 and Baseline (95% CI)
**Severity**					
**BEBSS**	35.9 (7.81)	30.3 (7.03)	5.6 (-7.39, -3.86)	31.1 (8.18)	4.79 (-6.18, -3.40)
**TBSA (%)**	16.3 (3.78)	10.9 (2.97)	5.4 (-8.14, -2.61)	13 (4.14)	3.3 (-4.68, -1.82)
**Blister**					
**Blister count**	7.7 (1.87)	3.7 (1.52)	4 (-6.74, -1.26)	2.3 (1.64)	5.3 (-0.87, -1.97)
**Blister area/BSA (%)**	4.9 (2.60)	2.9 (1.79)	2 (-4.02, -0.06)	2.3 (1.27)	2.6 (-5.59, 0.28)
**Pain and pruritus**					
**Pain score**	7.5 (1.52)	4.5 (1.79)	3 (-4.76, -1.24)	5.5 (2.49)	2 (-3.63, -0.37)
**Pruritus score**	7.2 (2.19)	5.2 (2.68)	2 (-3.76, -0.24)	5.3 (2.49)	1.8 (-3.52, -0.15)
**Quality of life**					
**QOLEB**	30.7 (6.77)	24.5 (5.39)	6.2 (-8.69, -3.65)	27.5 (9.89)	3.2 (-6.77, 0.43)

SD, Standard deviation; CI, confidence interval; BEBSS, The Birmingham Epidermolysis Bullosa Severity; TBSA, Total body surface area affected by epidermolysis bullosa; BSA, Body surface area; QOLEB, Quality of life in epidermolysis bullosa questionnaire

Supplemental Figures

Supplemental Figure 1.

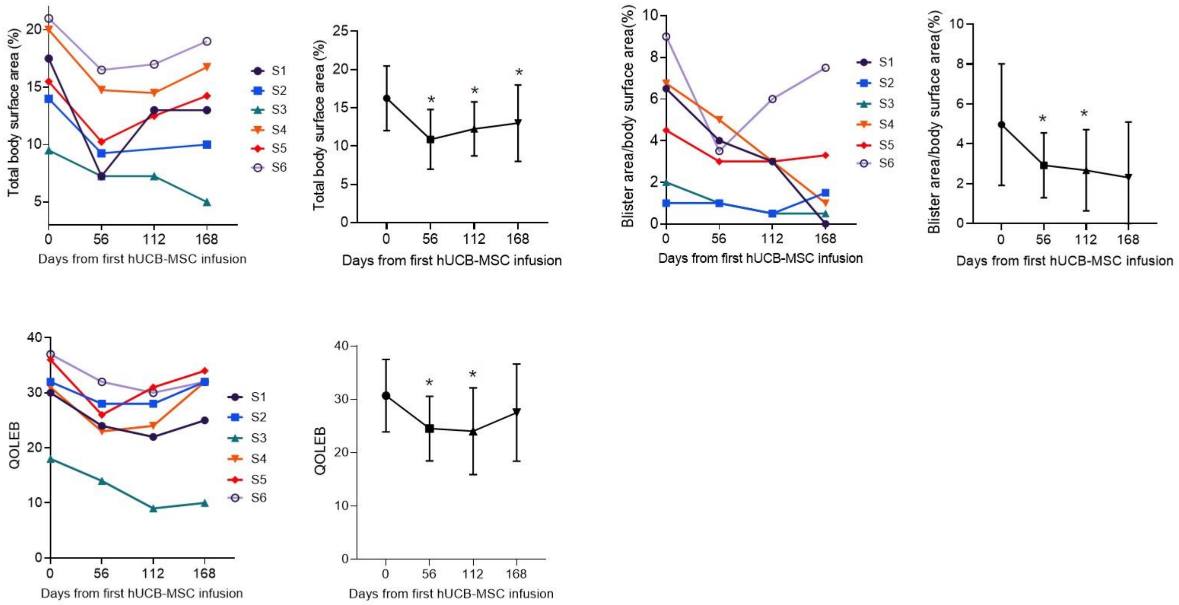

Supplemental Figure 1. Clinical responses over time. Time course of changes in total body surface area affected by EB (%), blister area per body surface area (%), and quality of life assessed by quality of life evaluation in epidermolysis bullosa (QOLEB) was assessed throughout the trial. For each parameter, a graphical representation of mean score per visit with range per visit was added. Two-tailed Student's t-test was performed for all the comparisons (n=6). *$P < 0.05$. S, subject.

Supplemental Figure 2.

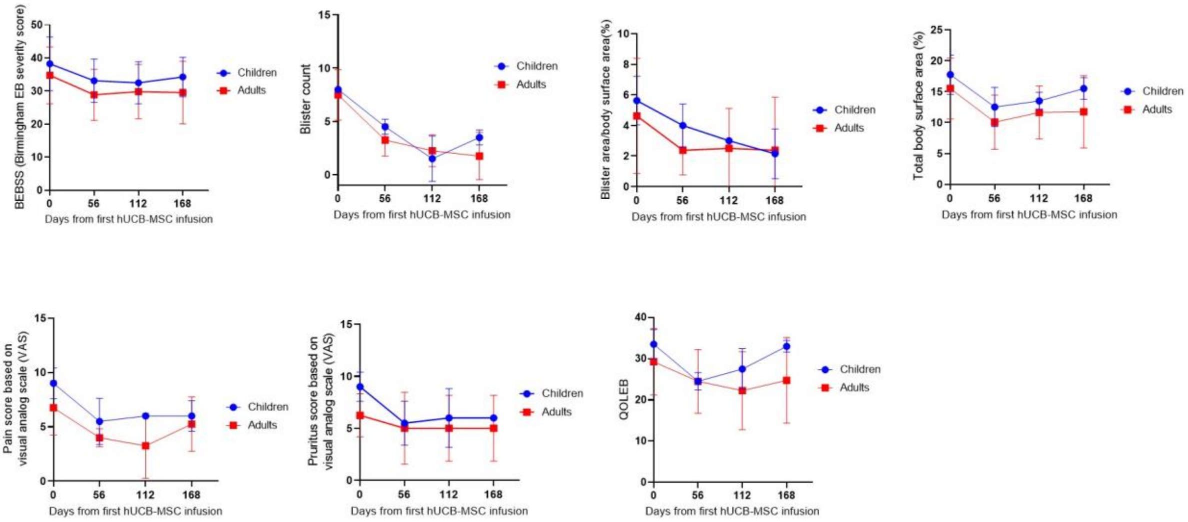

Supplemental Figure 2. Clinical responses over time. Time course of changes in secondary outcome measures in each age subgroup (children vs. adults) was assessed throughout the trial. For each parameter, a graphical representation of mean score per visit with range per visit was added. Two-tailed Student's t-test was applied for all the comparisons (n=2 for children, n=4 for adults).
